# Comparison of Serological Methods for Tick-Borne Encephalitis Virus-Specific Antibody Detection in Wild Boar and Sheep: Impact of the Screening Approach on the Estimated Seroprevalence

**DOI:** 10.3390/v15020459

**Published:** 2023-02-06

**Authors:** Gabrielle Trozzi, Nadjah Radia Adjadj, Muriel Vervaeke, Severine Matthijs, Charlotte Sohier, Nick De Regge

**Affiliations:** 1 Unit of Exotic and Vector-Borne Diseases, Sciensano, Groeselenberg 99, 1180 Brussels, Belgium; 2 Agency for Nature and Forests, 1000 Brussels, Belgium; 3 Viral Reemerging, Enzootic and Bee Diseases, Sciensano, Groeselenberg 99, 1180 Brussels, Belgium

**Keywords:** tick-borne encephalitis virus, diagnostic, ELISA, seroprevalence

## Abstract

Tick-borne encephalitis virus (TBEV) is a flavivirus transmitted by ticks. Serological screenings in animals are performed to estimate the prevalence and distribution of TBEV. Most screenings consist of a primary screening by ELISA, followed by confirmation of positive samples by plaque reduction neutralization tests (PRNTs). In this study, 406 wild boar sera were tested with 2 regularly used commercial ELISAs for flavivirus screening in animals (Immunozym FSME (TBEV) IgG All Species (Progen) and ID Screen West Nile Competition (Innovative Diagnostics)) and PRNTs for TBEV and USUTU virus. The results showed that the Immunozym and IDScreen ELISAs had low relative sensitivities of 23% and 20%, respectively, compared to the PRNT results. The relative specificities were 88% and 84% due to cross reactions with USUTU virus-specific antibodies. The minimal TBEV prevalence in our sample set was 8.6% when determined by PRNT. When the screening approach of ELISA testing followed by PRNT confirmation was applied, a TBEV seroprevalence of only 2.0% and 1.7% was found. The suboptimal performance of the ELISAs was confirmed by testing sera collected from experimentally TBEV-infected sheep. While the PRNT detected TBEV specific antibodies in 94% of samples collected between 7 and 18 days post-infection, the ELISAs classified only 50% and 31% of the samples as positive. Both routinely used ELISAs for TBEV antibody screening in animal sera were shown to have a low sensitivity, potentially leading to an underestimation of the true prevalence, and furthermore cross-react with other flavivirus antibodies.

## 1. Introduction

Tick-borne encephalitis virus (TBEV) belongs to the family of the Flaviviridae, and is closely related to flaviviruses such as the West Nile virus (WNV) and USUTU virus (USUV) [1]. TBEV causes tick-borne encephalitis (TBE) in humans, a viral infection that causes mild or moderate febrile illness, and in some cases leads to fatal encephalitis [2]. TBEV was previously divided into three subtypes, but due to its genomic diversity, a new classification of TBEV into seven subtypes has recently been proposed [3].

TBEV is an arbovirus transmitted by hard ticks. In Europe, *Ixodes ricinus* is the principal arthropod vector. *Ixodes ricinus* is a three-stage parasite (larvae, nymphs and adults) that feeds for several days at each stage on a mammalian host [4]. This species completes its life cycle in 2 to 6 years. It may be infected by TBEV at each stage and become a carrier for the rest of its life. Ticks mainly become infected by the virus by feeding on infected animals or by co-feeding (feeding of infected and uninfected vectors in spatiotemporal proximity [5]). Virus transmission to the next life stage occurs by transovarial and transstadial transmission [6]. Nymphs represent the most important life stage in transmission and infection. This stage is less host-specific, so more species can be infected, and nymphs are present in larger numbers than adult ticks [7].

Some rodent species act as major reservoirs of TBEV, since they have a high viremia and may transmit the virus to ticks that take a bloodmeal [8]. Other species, such as roe deer, cows, etc., also play a role in the transmission of the virus. Although their viremia is low, they may act as a support for transmission via co-feeding. These species produce antibodies against TBEV after infection. Birds seem to play a role in the spread of the virus over long distances [9]. Humans can also be infected by TBEV, but they are dead-end hosts that do not play a role in further transmission of the virus [7]. 

In Belgium, the TBEV seroprevalence was examined in different animal species. TBEV-specific antibodies have been detected in wild boar, roe deer, domestic dogs, cattle and sheep [10,11,12,13,14,15,16]. Depending on the species, the detected seroprevalence varied between 0.35% (dogs [12]) and 12.4% (roe deer [14]). Recently, our laboratory reported a TBEV seroprevalence of 0.42% in sheep and 9.27% in wild boar [15]. In 2020, for the first time, three autochthonous cases were identified in humans [17]. Ticks have also been screened, but so far, TBEV has not been detected [15]. This is probably due to the fact that TBEV is only present in ticks in very small focus areas [10]. 

In other countries, similar studies have also been conducted to estimate the TBEV seroprevalence and to obtain insight into the distribution of the virus in the country of interest [18]. Different species involved in the TBEV transmission cycle have been used in these screenings based on their capacity to develop persistent TBEV antibody responses, e.g., domestic sheep [19], wild red foxes [20], wild boars [21], wild cervids [22] and rodents [9]. Recently, bison [23] and voles [24,25] have been used for TBEV screenings in Poland. The seroprevalence varied between 3.9% (in voles) [24] to 63.5% (in bison) [23]. These results show the importance of finding a species that is representative of virus circulation and that allows for monitoring over the years. Wild boars seem to be a good animal species to obtain an understanding of TBEV incidence. They play an important role in the maintenance of the tick population, have a limited home range and show a strong seroconversion [26].

A thorough literature search showed that the majority (83%) of seroprevalence studies in animals use a similar methodology. Most often, serum samples are first screened with an ELISA kit, since this is easy to perform and delivers relatively cheap and fast results. Afterwards, the positive samples are subjected to a virus neutralization test, which is considered as the golden standard test for flavivirus antibody detection, to eliminate potential false positive results. From 38 identified screening studies of flavivirus antibodies in animals, 19 used the Immunozym FSME IgG All Species ELISA (Progen, Heidelberg, Germany) [12,14,15,19,20,21,23,24,25,26,27,28,29,30,31,32,33,34,35], 10 used the ID Screen WNV Competition ELISA Kit (Innovative diagnostics, Montpellier, France) [13,33,36,37,38,39,40,41,42,43] and 9 used other ELISA kits [9,22,44,45,46,47,48,49,50] as a first screening. Based on this literature study, it was decided to evaluate the performance of the Immunozym ELISA and the IDScreen ELISA in more details.

The Immunozym ELISA was designed to detect anti-TBEV antibodies. The relative performance of the Immunozym ELISA to detect TBEV antibodies compared to the PRNT was previously determined in dogs [35] and in foxes [20]. In dogs, the relative sensitivity of the Immunozym ELISA was 84.8% and the relative specificity was 99.4% [35]. In foxes, results were less accurate, with a relative sensitivity of 42.3% and a relative specificity of 98.9%. This low relative sensitivity was attributed to the low titer of antibodies present in foxes [20]. The Immunozym ELISA kit was also tested on human sera with a known status. In that test, the sensitivity was 83% and the specificity was 49%. The low specificity was probably due to cross-reaction with other flaviviruses [51]. The ID Screen ELISA is intended for the detection of WNV-specific antibodies, but it is now marketed for the detection of anti-flavivirus antibodies based on its known cross-reactivity with antibodies from other flaviviruses [51]. The performance of the IDScreen ELISA was evaluated in horses [52] and birds [53,54] for WNV-specific antibodies with satisfactory results. Although regularly used in TBEV screening, its performance to detect TBEV antibodies has not yet been evaluated.

The aim of this study was to evaluate the ability of these two ELISA kits compared to PRNT to detect TBEV antibodies in sera from animals that are frequently used in screenings for TBEV, such as wild boar and sheep. This, furthermore, allows us to evaluate the influence of the screening approach on the predicted TBEV prevalence.

## 2. Materials and Methods

### 2.1. Samples

- Wild boar sera

Wild boar sera (n = 406) were selected from a set of 886 serum samples that had previously been used to determine the TBEV seroprevalence in Flanders, Belgium [15]. All samples for which sufficient serum remained to perform all serological tests were used. The samples originated from hunted wild boar in 2019–2020, and sera were stored at −20 °C until testing.

First, 100 µL of each serum was treated with kaolin (Sigma-Aldrich, St. Louis, MO, USA) to remove nonspecific inhibitors [55]. Therefore, one volume of serum was mixed with four volumes of a 25% solution of kaolin in PBS. The mix was vortexed and incubated for 30 min at room temperature, followed by centrifugation for 10 min at 3500 rpm. The supernatant was used for testing.

- Domestic pig sera

Pig sera (n = 139) were collected at 4 commercial Belgian farms and were stored at −20 °C until testing. Since these pigs had no outdoor access, their sera were considered negative for TBEV. These were used to determine the cut-off for positivity of the TBEV PRNT test.

- Sheep sera

Sheep sera (n = 60; 36 from TBEV-infected sheep and 24 from control sheep) from an in vivo infection experiment were used (Adjadj et al., in preparation). The experiment was approved by the ethical committee of Sciensano (approval number: 20200515-01) and was conducted in BSL3 animal facilities. All animals were tested for the presence of antibodies against Flaviviruses before the start of the experiment and were negative. Sheep (n = 26) were divided in two groups. The first group of 16 sheep was infected by intradermal inoculation of TBEV (10^5^ TCID_50_/animal), while a second group of 10 sheep was mock-infected with MEM and kept as a control group. Blood was collected from two sheep of the control group at the day of infection (0 dpi). At 1, 2, 3, 5, 7, 10, 14 and 18 dpi, blood was collected from 5 and 3 sheep from the infected and control group, respectively. Blood from all remaining animals was collected at 14 (4 animals) and 18 (2 animals) dpi. Before testing, all samples were heat-inactivated for 30 min at 56 °C.

### 2.2. ELISAs

Sera were tested using the Immunozym FSME IgG All Species ELISA (Progen, Heidelberg, Germany) and the ID Screen WNV Competition ELISA Kit (Innovative diagnostics, Montpellier, France) according to the manufacturer’s instructions. 

The Immunozym FSME IgG All Species ELISA is an indirect ELISA. Wells are coated with inactivated TBE virus and a protein G peroxidase conjugate is used for the detection of anti-TBE-IgG antibodies. The results are expressed in Vienna Units per milliliter (VIEU/mL). According to the manufacturer’s instructions, a concentration <63 VIEU/mL is considered as negative, between 63 to 126 VIEU/mL as borderline and >126 VIEU/mL as positive. For our analysis, borderline results were considered as positive.

The ID Screen WNV Competition ELISA is a competition ELISA. Wells are coated with a purified extract of West Nile virus, and a peroxidase-labeled anti-pr-E (envelope protein) antibody is used to check for competing anti-WNV antibodies. The results were expressed as a % S/N value (OD sample/OD negative control). A % S/N >50% was considered as negative, between 40 and 50% as doubtful and <40% as positive. For our analysis, the doubtful results were considered positive.

### 2.3. Plaque Reduction Neutralization Tests

Plaque reduction neutralization tests (PRNTs) were performed in a rapid fluorescent foci inhibition test (RFFIT) format against TBE and USUTU viruses using Vero cells grown in 96-well plates. Fivefold sera dilutions of the samples and positive and negative controls were made in the first row of the 96-well plate. Then, each sample was further diluted (to 1/80) by a twofold serial dilution in DMEM (Gibco^TM^ Thermo Fisher Scientific, Waltham, MA, USA supplemented with 5% fetal bovine serum (Life Technologies, Carlsbad, CA, USA).

A total of 50 µL of each serum dilution was then mixed with 50 µL of DMEM containing between 20 to 50 PFU of either TBEV or USUV, and the mix was incubated for 1 h at 37 °C and 5% CO_2_. Thereafter, the serum/virus mix was added to 90% confluent Vero cells and incubated for 27 h for USUV, and for 66 h for TBEV, at 37 °C and 5% CO_2_. Then, the culture medium was removed and the wells were washed with 150 µL of phosphate-buffered saline (PBS). Cells were fixed with 75 µL of cold 100% methanol for 20 min at −20 °C. Afterwards, the methanol was removed and plates were dried at −20 °C for at least 24 h. 

Immunofluorescent staining was performed to visualize viral antigens. Primary mouse-anti-TBEV-NS1 and anti-WNV-NS1 (based on its tested cross-reactivity with USUV) antibodies (R&D systems, Bio-Techne Ltd., Minneapolis, MN, USA) were used for TBEV and USUV antigen detection, respectively, and a secondary Alexa Fluor 488 goat anti-mouse immunoglobulin G (IgG) antibody (Life Technologies, Carlsbad, CA, USA) for visualization. All antibodies were used at a 1/200 dilution and incubated for 1 h at 37 °C. In the final washing step, which lasted 3 min, Hoechst (10 µg/nL) was added to the PBS to visualize DNA. 

The number of plaques was counted under the fluorescence microscope. Wells with less than 50% of the plaques compared to the control wells were considered as neutralized. Testing of negative pig sera was performed to determine the cut-off value (see below). Sera with a titer <1/10 (last dilution where the sample is neutralized) were considered as negative; sera with a titer ≥1/10 were considered as positive. A similar cut-off in PRNT for TBEV has already been used in the literature [11], and was also used for USUV [49,56].

Based on the obtained PRNT_50_ titers for both viruses, a final status was given to each sample. If no antibodies were detected in either PRNT, the status was negative; if a >= 4-fold difference in PRNT titers was obtained between both viruses, the status corresponding to the virus with the highest titer was accorded. If the difference between both PRNT titers was less than 4-fold, the status “Flavivirus-positive” was given.

### 2.4. Statistical Analysis

The interval of 95% confidence (CI) was calculated with the Wilson’s method on the Epitools website “https://epitools.ausvet.com.au/”(accessed on 30 November 2022).

To compare the performance of the tests which we used, the kappa test was used on the Epitools website (accessed on 23 January 2023). The interpretation of agreement was: ≤0  =  poor, 0.01–0.2  =  slight, 0.21–0.4  =  fair, 0.41–0.60  =  moderate, 0.61–0.80  =  substantial and 0.81–1  =  almost perfect concordance [57].

## 3. Results

### 3.1. Determination of the Cut-Off for Positivity of the TBEV PRNT

One hundred thirty-nine sera from indoor-housed pigs, thus considered as TBEV negative, were tested using the PRNT for TBEV.

**Figure 1:**
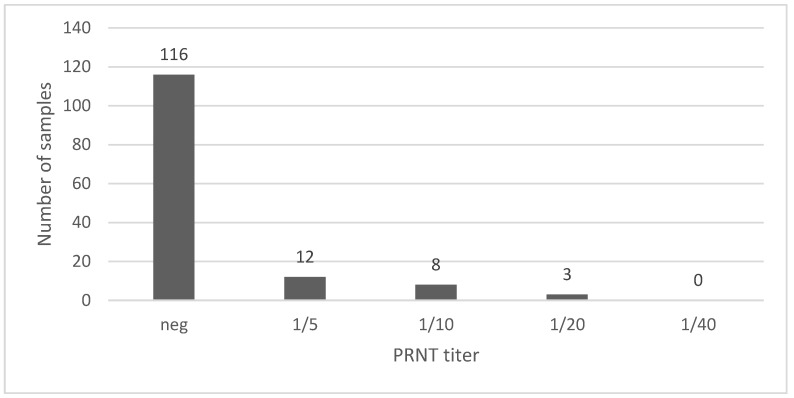
PRNT titers obtained in sera (n = 139) from commercial pigs without outdoor access.

Figure 1 shows that 92% (128/139, CI 95%: 86.4–95.5) of samples were either negative or scored a titer 1/5. Only 11 out of 139 samples had a titer ≥1/10. It was, therefore, decided that the cut-off for positivity would be fixed at 1/10, corresponding to a test specificity of 92%.

### 3.2. Detection of Neutralizing Antibodies by PRNT in Wild Boar

Four hundred and six kaolin-treated wild boar sera were tested using PRNTs for TBEV and USUV. In PRNT, 8.62% (35/406, CI 95%: 6.26–11.75) of sera were positive for TBEV-specific antibodies, 18.47% (75/406, CI 95%: 15–22.54)) tested positive for USUV-specific antibodies and 5.42% (22/406, CI 95%: 3.61–8.07) received the status of FLAVI positive, since less than a 4-fold difference was found between the two PRNTs. For further comparative analysis, only the PRNT TBEV-positive samples were considered as TBEV-positive, and the FLAVI-positive samples were considered as TBEV-negative.

### 3.3. Diagnostic Performance of ELISAs Compared to PRNT on Wild Boar Samples

**Figure 2:**
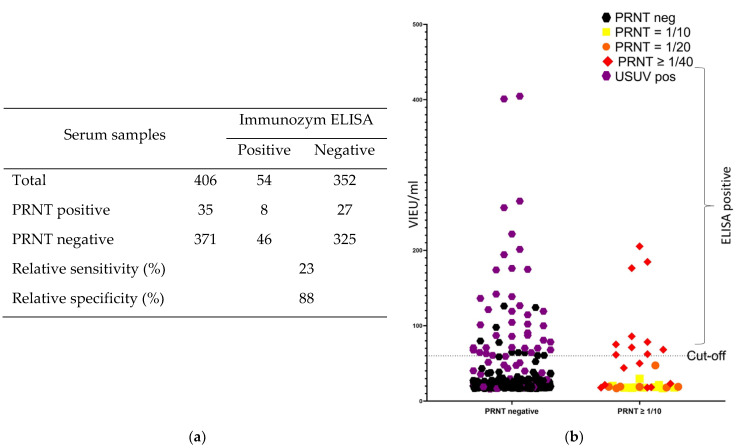
Relative diagnostic performance of the Immunozym ELISA compared to PRNT for detection of anti-TBEV-specific antibodies in wild boar sera. (**a**) Comparative table of the results obtained in ELISA to the golden standard. (**b**) Distribution of the ELISA results in function of the PRNT status and titer.

The samples from wild boar were tested by two commercial ELISA tests. In the Immunozym ELISA, only 8 of the 35 PRNT TBEV-positive samples were found to be positive, corresponding to a relative sensitivity of only 23% for this ELISA (Figure 2a). On the other hand, 46 of the 371 PRNT TBEV-negative samples were found to be positive by ELISA, corresponding to a relative specificity of 88%. The overall poor concordance between ELISA and PRNT results was also confirmed by a Kappa value of only 0.083, indicating a slight concordance. This low specificity is mainly due to the cross-reaction with antibodies against USUV. Out of the 46 false positive samples, 38 were identified in PRNT as USUV-positive (Figure 2b: purple), 5 had a FLAVI-positive status and the remaining 3 were negative in both PRNTs. Although a clear cross-reaction with USUV-specific antibodies was identified, this only occurred in some of the USUV-positive samples, since only 38 out of 75 USUV-positive samples were found to be positive by ELISA. 

The detection of TBEV-specific antibodies by the Immunozym ELISA seems to be correlated with the amount of antibodies present in the samples. Only samples with the highest titer (≥1/40) in PRNT were detected as positive by the ELISA (Figure 2b), although even some of these highly positive samples were missed.

**Figure 3:**
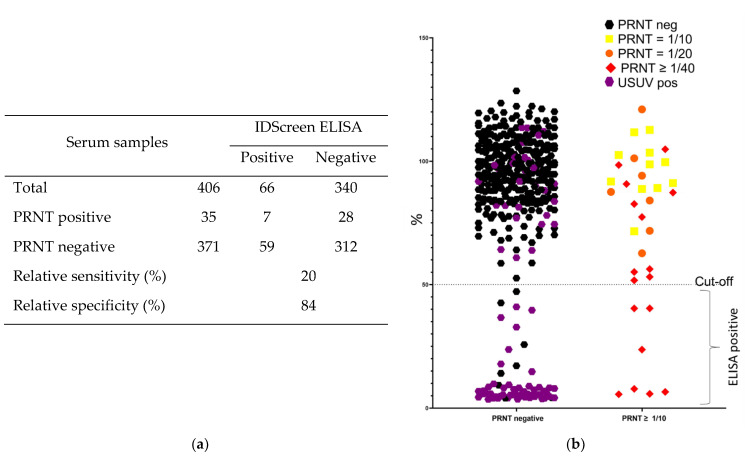
Relative diagnostic performance of the IDScreen ELISA compared to PRNT for detection of anti-TBEV-specific antibodies in wild boar sera. (**a**) Comparative table of the results obtained by ELISA to the golden standard. (**b**) Distribution of the ELISA results in function of the PRNT status and titer.

In the IDScreen ELISA, only 7 out of 35 PRNT TBEV-positive samples were identified as positive, corresponding to a relative sensitivity of 20% for TBEV (Figure 3a). In total, 59 of the 371 PRNT negative samples were detected as positive in this ELISA, resulting in a relative specificity of 84%. The overall poor concordance between the ELISA and PRNT results was also confirmed by a Kappa value of only 0.029, indicating a slight concordance. Out of the 59 samples identified as false positive, 51 were identified as USUV-positive by PRNT (Figure 3b: purple), 7 as FLAVI and 1 as negative. Cross-reaction with USUV-specific antibodies occurred in a major part of the USUV positive samples, since 51 out of 75 USUV-positive samples were found to be positive with the IDScreen ELISA.

Similarly to the results observed for the Immunozym ELISA, the TBEV-specific antibody detection by the IDScreen ELISA seems to be correlated with the amount of antibodies present in the samples. Only samples with high titers in PRNT (≥1/40) were detected as positive by ELISA (Figure 3b).

Since PRNT and ELISA results were available for all samples, it was possible to evaluate the impact of the screening approach on the predicted TBEV prevalence during an a posteriori analysis. If a screening had been conducted using only PRNT tests, the minimal TBEV prevalence in our sample set would have been estimated to be 8.62% (considering FLAVI-positive samples as TBEV-negative). In contrast, if the sample set had first been screened in ELISA followed by confirmation tests in PRNT, the seroprevalence would have been estimated to be 1.97% (8/406, CI 95%: 1.00–3.84) and 1.72% (7/406, CI 95%: 0.84–3.52), respectively, with a primary screening in the Immunozym ELISA or the IDScreen ELISA.

### 3.4. Diagnostic Performance of ELISAs Compared to PRNT in Samples from Experimentally Infected Sheep

To verify whether the suboptimal ELISA results obtained in wild boar sera are specific to this species, a similar analysis was conducted on serum samples collected from sheep which were experimentally infected with TBEV (Adjadj et al., in preparation).

First, 24 samples from control animals (not infected by TBEV) were tested by PRNT and both ELISAs to evaluate the specificity. All samples tested negative in all tests, demonstrating that the ELISA kits are specific when no cross-reactive antibodies are present due to a previous infection with another flavivirus. 

TBEV-specific antibodies were first detected at day 7 post-infection (p.i.) (4/5 sheep) by PRNT. All tested samples from later time points were PRNT-positive until the end of the experiment, at day 18 p.i. (Table 1).

In the Immunozym ELISA, first, TBEV antibody positive sera (3 out of 5) were only detected at day 10 p.i.. All samples were scored positive at 14 dpi. At 18 dpi, 1 out of 2 PRNT positive samples scored negative in this ELISA. 

Similar results were found with the IDScreen ELISA. The first TBEV-positive sera were also only detected at 10 dpi. At 14 dpi, 2 out of 4 serum samples were positive, while both PRNT positive samples collected at 18 dpi scored negative in ELISA. 

**Figure 4:**
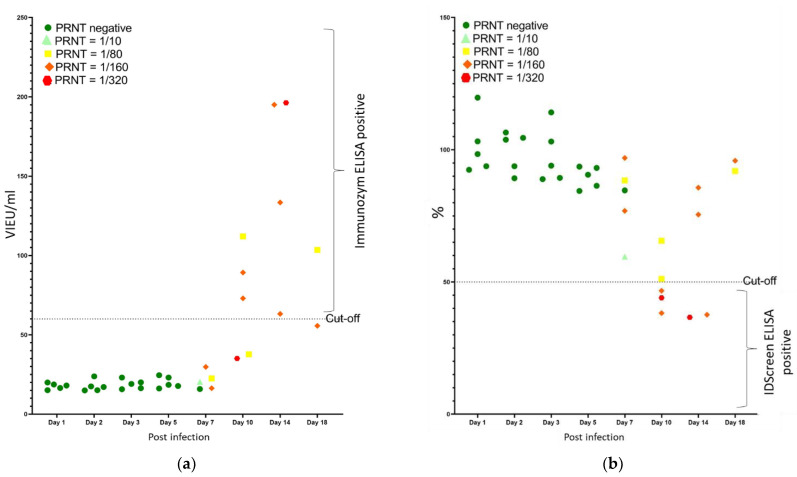
Quantitative ELISA results versus PRNT titers obtained in sera collected between 1 and 18 days post-experimental TBEV infection of sheep. Results were obtained with the Immunozym ELISA (**a**) and the IDScreen ELISA (**b**).

The analysis of the quantitative ELISA results compared to the PRNT titers (Figure 4a) showed that all negative samples in PRNT were correctly identified in the Immunozym ELISA, corresponding to a specificity of 100%. Starting from 7 dpi, only 50% of the PRNT positive samples were found to be ELISA positive, and multiple samples with a high titer in PRNT (up to 1/320) remained negative in ELISA. It should, furthermore, be mentioned that most of the samples which we considered positive in ELISA were only classified as doubtful based on the manufacturer’s instructions. 

The IDScreen ELISA gave similar results as the Immunozym ELISA. The specificity was 100%, as all negative sera in PRNT were negative in ELISA. For samples detected as positive by PRNT, only those with the highest titers (half of sera with a titer of 1/160 and all with a titer of 1/320) were detected as positive by ELISA. Figure 4b shows that even the samples that scored ELISA positive had S/N values close to the cut-off. If the doubtful samples of this kit had been considered negative (i.e., S/N% = 40), only three samples would have been found positive. 

Overall, the results obtained in sheep sera confirm the results obtained in wild boar sera and highlight the low sensitivity of the ELISA kits for TBEV antibody detection.

## 4. Discussion

TBEV monitoring in different animal species has previously been used to obtain insight into the presence and distribution of the virus in specific regions and countries. It has allowed for the discovery of TBEV foci in regions considered as non-risk areas. Previous studies [16,35,51,58] on TBEV prevalence have shown that both the animal species and the diagnostic methods used can impact the results. The availability of reliable tests with good sensitivity and specificity is crucial for a correct estimation of the prevalence. Virus neutralization tests, mostly performed as PRNTs for flaviviruses, are considered to be the golden standard serological method, but they are not often used for screening of a high number of samples due to the labor-intensiveness of the manipulations, the relatively long time period to obtain results, the high costs and the need to work in laboratories with high biosafety levels. Therefore, PRNTs are mostly replaced by ELISAs, which are more rapid, cost less and are commercially available. Data on the diagnostic performance of these ELISAs used for TBEV-specific antibody detection are, however, scarce. 

We, therefore, first compared the performance of two frequently used commercial ELISA kits relative to PRNT reference tests on a sample set of 406 wild boar sera. The sensitivity of both ELISAs to detect TBEV-specific antibodies in wild boar sera was found to be very low (<30%), and only the sera with the highest PRNT titers were found positive in the ELISAs.

The low sensitivity of the Progen ELISA for TBEV detection in wild boar sera was also previously reported in a study of Roelandt et al. [11]; however, that study analyzed only a limited number of samples. Out of seven PRNT positive samples, four were found to be positive by ELISA. In a study on foxes by Haut et al. [20], similar results were obtained. They hypothesized that the low sensitivity was due to the dilution of their samples and/or the decrease in antibody levels in the period between infection and sampling. Since the tested ELISA kits only detected those samples with the highest PRNT titers, it seems that they are only useful for monitoring shortly after infection, since flavivirus antibodies decrease afterwards. However, for wild animals, the duration of the antibody response is unknown, and no information on the persistence of these antibodies after infection are available [20].

Results indicating a low sensitivity were also obtained in the study of Klaus et al. [58], who studied goat sera using two Progen ELISAs. They showed that the Immunozym FSME IgM kit (not available anymore) had a better sensitivity (89%) than the Immunozym FSME IgG all species kit (57%) which we used. Since they used sera from vaccinated goats, they hypothesized that the lower sensitivity was due to a predominant IgM response early after vaccination. As neither the time since infection nor the IgG/IgM ratio in the wild boar was known, it is difficult to judge to what degree this influenced our results, but we estimated that this cannot be the major explanation for the observed low sensitivity. Klaus et al. [58] also proposed that the cut-off value of the Progen ELISA be raised for analysis of pig sera to avoid false positive results. If this had been applied to our study, the specificity would indeed have been increased, but it would have further decreased the sensitivity, as some positive samples were already at the cut-off.

Our samples were obtained from hunted wild boar, and the quality of the sera was not optimal. Some sera showed extensive hemolysis, bacterial contamination or induced toxic effects on cells, all of which can affect the detection of antibodies. A kaolin treatment was, therefore, performed to optimize the quality of the samples and to remove non-specific inhibitors [59]. It has, however, been described that a kaolin treatment can also deplete IgG, IgM and IgA antibodies that are present in serum samples [60]. A study by Mann et al. [55] showed that kaolin treatment decreased the quantity of immunoglobulins by 40 to 60%. Therefore, to verify whether the low ELISA sensitivity found in wild boar sera was due to the sample quality and kaolin treatment, sheep samples of optimal quality were collected from experimentally infected animals and tested without previous treatment. Seroconversion was detected later in both ELISAs than in PRNT in these sheep, and only a part of the PRNT positive samples with high titers were positive in ELISA. The results obtained for the sheep sera confirmed the results from the wild boar sera, namely that both ELISAs lack the sufficient sensitivity to detect TBEV-specific antibodies, and thus showed that the low sensitivity was not solely the consequence of the species studied nor of the sample quality. Sheep have already been used for TBEV monitoring in different studies [33,61,62], and although only 18 sheep sera were tested using 3 methods in a 2020 study by Khbou et al. [33] investigating the TBEV presence in Tunisia, the results demonstrated the absence of agreement between ELISA and PRNT results. 

Besides sensitivity, the specificity of the ELISAs was also evaluated using the wild boar and sheep sera. In the sheep samples, which were free of other flavivirus antibodies, the specificity was 100%, demonstrating the intrinsic high specificity of the ELISA tests. The specificity for the wild boar sera was, however, lower: 88% and 84% for the Immunozym and the IDScreen ELISA, respectively. The suboptimal specificity was due to cross-reactivity with USUV-specific antibodies. The presence of USUV-specific antibodies in Belgian wild boar was not unexpected. The virus spread in European countries in 2012 and reemerged in 2016, after which it continued circulating at a low level in Belgium [63]. USUV-specific antibodies were also detected in wild boar in France between 2009 and 2014, where a seroprevalence of 8.0% was found in this species [64]. These results highlight that further efforts should be made to develop more specific ELISA kits for the different flaviviruses in order to prevent an incorrect interpretation of obtained diagnostic results. In our study, we did not perform PRNT tests for West Nile virus since this virus has never been reported in Belgium, but in endemic areas or areas where WNV is emerging, this is another flavivirus for which a possible cross-reaction needs to be considered during interpretation of ELISA results. 

Our data also allowed us to evaluate the potential impact of the screening approach on the estimated TBEV seroprevalence. In most TBEV screenings reported in the literature, sera are first screened by ELISA, and afterwards, positive samples are tested by PRNT for confirmation and identification of false positive results. Our results showing the low sensitivity of both ELISAs for TBEV antibodies in wild boar and sheep sera indicate that many positive samples will be missed by this approach, resulting in an important underestimation of the TBEV seroprevalence. Using our sample set, the TBEV seroprevalence in wild boar would be estimated to be around 2% using the approach of ELISA testing followed by PRNT confirmation, but it was almost 9% when directly using the golden standard method. The underestimation of the TBEV prevalence resulting from an ELISA screening can have a major impact on public health, as it can lead to an underestimation of the human infection risk and the preventive measures that should be deployed to inform the public about this emerging disease. This also means that in areas with low expected TBEV prevalence in wildlife, potential positive cases are not detected in wildlife disease monitoring. This result advocates for the preferential use of PRNT tests during TBEV screenings and/or to optimize TBEV ELISAs. Furthermore, it also highlights that one should be cautious when comparing seroprevalence data between studies or trends in seroprevalence over time. This only seems to be possible when a similar screening approach is used and the same species is targeted. It is also important to bear in mind that cross-reactions between flaviviruses exist, and that it is possible that part of the ELISA positive results are due to the detection of antibodies against another flavivirus. Additionally, actual co-infections with multiple flaviviruses can occur [65], adding further complexity to the interpretation of serology results.

## 5. Conclusions

In conclusion, this study shows that two ELISAs which are routinely used to assess TBEV seroprevalence in animal species have a low relative sensitivity compared to PRNT in wild boar and sheep sera, leading to an underestimation of the seroprevalence in these populations. It would also be useful to evaluate the diagnostic performance of these ELISA kits in other species. The specificity of both ELISAs was also shown to be suboptimal due to cross-reactions with antibodies against others flaviviruses. It is critical to keep this in mind during serological TBEV screenings and it highlights that attention should be paid to the used serological methods, as well as the screening approach, when comparing seroprevalences over time or between studies. The reported low sensitivity and specificity of the ELISAs furthermore indicates that further efforts should be made to develop highly sensitive and specific kits for the different circulating and/or emerging flaviviruses.

## Figures and Tables

**Table 1:** TBEV-specific antibody detection by ELISA and PRNT results for sera collected from experimentally TBEV-infected sheep (#: samples).

Days post-infection	1	2	3	5	7	10	14	18
Available samples	5	5	5	5	5	5	4	2
# pos in PRNT	0	0	0	0	4	5	4	2
# pos in Immunozym ELISA	0	0	0	0	0	3	4	1
# pos in IDScreen ELISA	0	0	0	0	0	3	2	0

## Data Availability

The data are included in the manuscript.
